# A self‐correcting method for improving the precision of beam blocks

**DOI:** 10.1120/jacmp.v2i3.2603

**Published:** 2001-09-01

**Authors:** Ivan A. Brezovich, Karen S. Sparks, Jun Duan

**Affiliations:** ^1^ Department of Radiation Oncology University of Alabama at Birmingham 619 South 19th Street Birmingham Alabama 35233

**Keywords:** radiation therapy, beam blocks, custom blocks, Cerrobend

## Abstract

A technique for manufacturing precise custom blocks is described. Using the tracing stylus of the mold making machine, reference markers are cut into the lateral borders of the polystyrene mold after the cavities for the blocks have been made. These markers are aligned with the central ray cross hair of the shadow tray when the blocks are mounted on the tray. The ability of the technique to enhance precision has been verified in laboratory tests by intentionally introducing small imperfections into a mold making machine and checking the positional accuracy of the mounted blocks. The clinical performance has been tested by evaluating 47 check films of blocks for 16 randomly selected patients. The average positional error of individual blocks, projected to the isocenter, was less than one mm. The average time needed to cut the reference markers was 25 seconds. Implementing the technique required only minor modifications of a commercial mold making machine.

PACS number(s): 87.53.–j, 87.56.–v

## INTRODUCTION

Modern radiotherapy, especially conformal therapy, puts even higher demands on the accuracy of radiation fields. To obtain the required accuracy, accelerators and simulators must be precisely built and aligned. The AAPM Radiation Therapy Committee Task Group 40^1^ (TG 40) report recommends x‐ray jaw position readouts to be accurate within 2 mm. Although the TG 40 report does not specify tolerances for custom blocks, it does emphasize the importance of accuracy. Considering that such blocks define field edges, it seems that they should be kept to the same standards as x‐ray jaws, i.e., their shadow in a plane through the isocenter should agree with the prescribed block outline within 2 mm or better.

**Figure 1:**
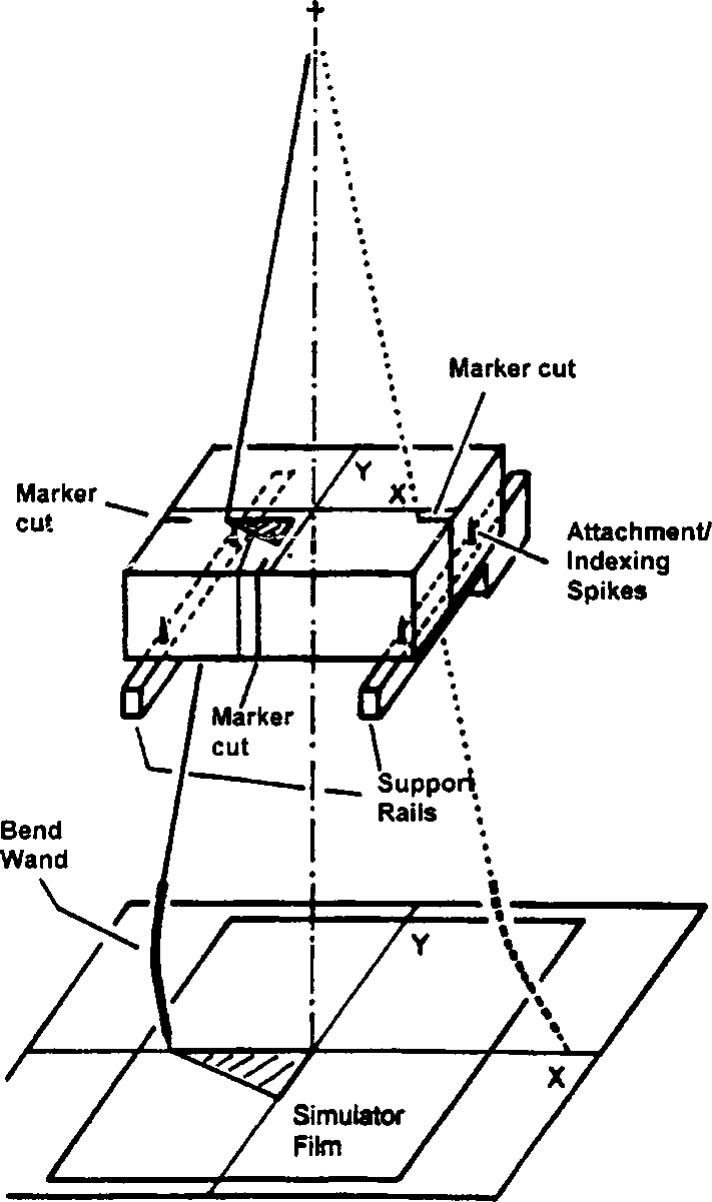
A bent tracing wand causes the cavity for the Cerrobend block to be shifted from the position drawn on the simulator film. However, the cavity is accurately positioned with respect to a coordinate system defined by reference markers cut into the lateral borders of the mold.

To meet these stringent demands, blocks must satisfy two criteria. First, their geometric shape must precisely match the outline of the simulation film. Second, blocks need to be positioned accurately within the radiation field. Methods and devices for manufacturing custom blocks have been described in the literature.^2^ ^,^ ^3^ Briefly, blocks are made by pouring a low melting alloy, e.g., Lipowitz's metal, often referred to as “Cerrobend” (Cerrobend® is a trademark of Cerro Copper and Brass Company, Belfonte, PA), into a mold of polystyrene foam. The mold, in turn, is made with the aid of a hot wire cutter reproducing the geometry of the simulator (Fig. 1). Experience and care in making the molds, like cutting slowly to prevent excessive deflection of the hot wire, and tracing slightly inside the block contour on the simulator film to compensate for the width of the cut, generally lead to precise beam blocks. To aid in the precise positioning of the blocks on the shadow tray, mold making machines are equipped with spikes or other devices that leave indexing marks on the styrofoam molds. These marks are aligned with corresponding markers on the shadow tray while the metal blocks are affixed to the tray. Finally, the mold is broken away, leaving only the blocks in place.

A drawback of the conventional block mounting method is that even small misalignment or imprecision of the mold cutting machine or the tracing wand is translated via the indexing system into imprecisely mounted blocks. A method for repositioning the blocks to correct for such errors has been reported in the literature.^4^ In this paper, an alternate technique that automatically corrects for small equipment imprecisions is suggested. The modifications of a commercial mold making machine required by the technique are described. The efficacy of the technique is evaluated in laboratory and clinical tests.

## METHODS AND MATERIALS

### Rationale for the self‐correcting technique

**Figure 2:**
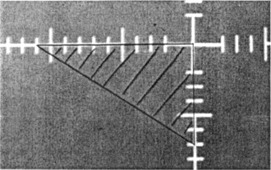
Simulator film with outlines of a beam block. Two sides of the triangular block are lying on the film axes.

To understand the self‐correcting features of our technique, we assume that a Cerrobend block is to be made according to an outline drawn on a given simulator film (Fig. 2). A styrofoam blank for the mold is placed on top of the support assembly of the mold cutting machine. The blank is pushed against the support rails so that the attachment spikes dig into the foam, holding it firmly in place (Fig. 1). We further assume that the mold making machine is accurately built and well aligned. This implies that the coordinate axes of the mold support assembly are parallel to the corresponding axes of the light box (film coordinate system). The main machine axis passes through the origins of both coordinate systems, is perpendicular to the film plane and the support assembly plane, and contains the pivot point of the tracing wand. Finally, we assume that the tracing wand is slightly bent. Because of this imperfection, the wand no longer accurately projects the film outline onto the styrofoam mold. Although the geometric shape of the cavity matches the simulator film, the cavity is shifted in relation to the coordinate axes of the support assembly. Hence, if the Cerrobend block is mounted on the tray using for alignment the spike imprints from the support assembly, the positioning error of the cavity is propagated.

**Figure 3:**
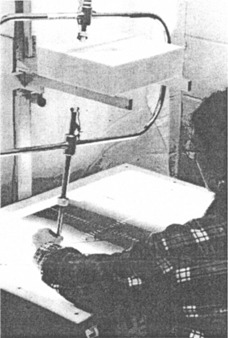
After the cavity for the block has been cut, reference markers are being cut into the lateral borders of the styrofoam mold.

**Figure 4:**
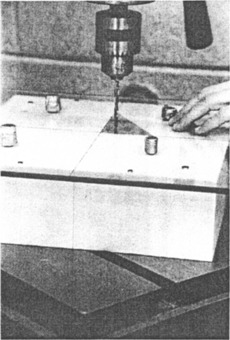
The central axis cross hair of the shadow tray has been aligned with the marker cuts on the styrofoam mold. Tray and mold are held temporarily in position by four auxiliary screws while holes are drilled through the plastic into the metal block for permanent attachment with self‐tapping sheet metal screws.

Our method requires the technician to cut three indexing markers into the borders of the styrofoam mold, carefully tracing the *x* and the *y* axes on the simulator film (Fig. 1, dotted wand, and Fig. 3). Being equally affected by the bent wand, these indexing cuts are shifted with respect to the coordinate system of the support assembly by the same distance as the cavity. The position of the cavity is therefore correct in relation to a coordinate system defined by these cuts. Hence, mounting the Cerrobend block while keeping these cuts aligned with the cross hair marking central ray on the shadow tray (Fig. 4), eliminates the detrimental effect of the bent wand. During the initial phases of the project, we relied on our machine shop to provide us with trays having the required cross hair. Such trays are now available from commercial suppliers.

Similar error elimination is achieved if the support assembly for the polystyrene mold is slightly shifted or rotated with respect to the coordinates of the light box. If we use a perfectly straight tracing wand, it produces index cuts that are accurate projections of the film coordinate system onto the mold. Hence, if the blocks are mounted on the tray using the index marks, their position matches the simulator film. If, as an additional imperfection, a slightly bent wand is used, cavity position and indexing marks are equally affected, leading again to an accurately mounted block.

### Modifications of a commercial block cutting machine

Figures 1 and 3 show a commercial mold cutting machine (Huestis Styroformer Model SF2, Huestis Medical, Bristol, RI) that was modified to allow implementing our self‐correcting technique. The key change consisted of moving each of the support rails for the styrofoam blank medially by about 5 mm. This shift causes the blank to protrude beyond the rails, providing space for the index marks. Because of the reduced clearance between the rails, the lower portion of the rails had to be cut away to prevent the hot wire from colliding with the rails when wide blocks were made. Reducing the original rail height of 50.8 mm (2”) to 25.4 mm (1”) had no noticeable effect on strength or rigidity of the mold cutter. Maintaining the original spacing between the support rails and using slightly wider styrofoam blanks would have been another option, but it would have increased the cost of the blanks. We also replaced the original tracing rod with one that was equipped with a stainless steel tip (Mick Radio‐Nuclear Instruments, Inc., New York, NY). Although the plastic tip of the commercial block maker contained a metal inset for improving rigidity, we preferred the even greater rigidity and ruggedness of steel. As a final modification we deepened the grooves marking the coordinate axes on the light box with the aid of a scribe. The deeper grooves provided positive guidance for the tracing tip when the index marks were cut.

## RESULTS

### Laboratory tests

**Figure 5:**
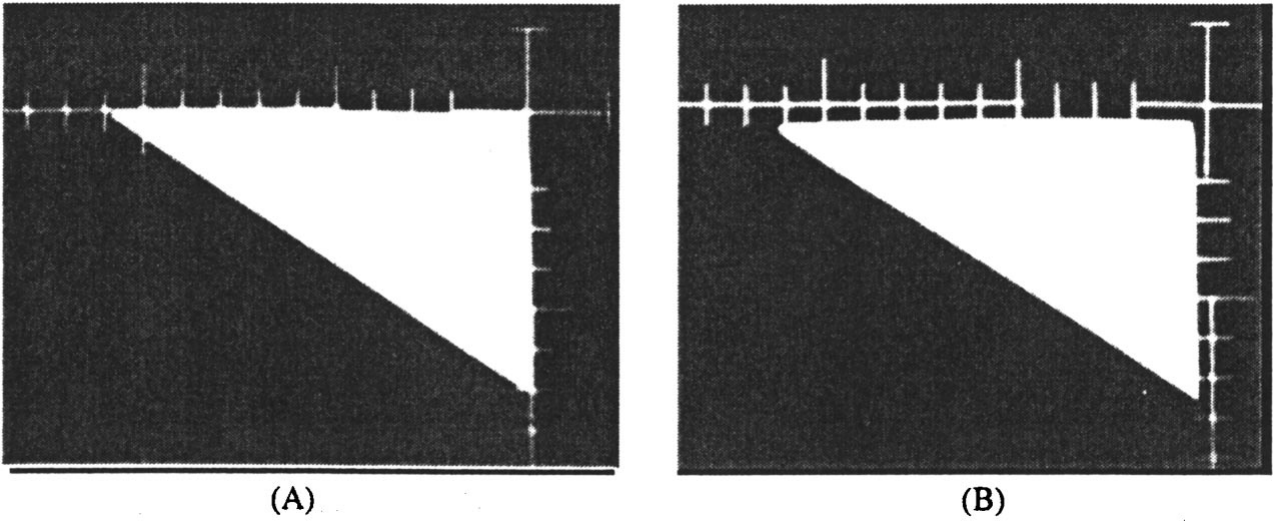
Simulator films of a beam block manufactured using a (intentionally) bent tracing wand. (A) The block made according to the self‐correcting technqiue accurately matches the simulator film. (B) When the conventional manufacturing technique is used, the defective wand leads to an imprecisely mounted beam block. Note that the two sides of the block are no longer located on the film axes.

To test the effectiveness of the self‐correcting technique, we intentionally bent the tracing stylus of our otherwise precise mold cutter, so that the position of the tracing tip deviated by 7 mm from its original position. We then made a Cerrobend block using our technique and, for comparison we also made one using the conventional method. The outline on the simulator film required the block to have the shape of a right triangle, with the two sides lying on the film axes (Fig. 2). After the cavity for the Cerrobend block had been made, we cut indexing marks into the borders of the $25.4\times 25.4\;{\text{cm}}^{2}(10^{\prime \prime }10^{\prime \prime })$ styrofoam blank as required by our technique (Fig. 3). The styrofoam blank was placed on a cold plate and the cavity filled with Cerrobend. After the Cerrobend had cooled, the styrofoam mold was turned over, and a shadow tray was placed on top of the blank while carefully aligning the central ray cross hair on the tray with the marker cuts (Fig. 4). Auxiliary sheet metal screws inserted through existing holes in the tray and driven into the foam kept the two pieces temporarily in place. We then drilled holes through the tray into the Cerrobend, and used self‐tapping sheet metal screws to hold the block securely in place. The auxiliary screws were removed, and the styrofoam mold broken away. A check film (Fig. 5A) shows that the block accurately matched the simulator film. Our technique automatically corrected for the flaw in the tracing wand.

To make a block according to the conventional technique, the support rails for the styrofoam blank were moved back to their original positions at the 10” mark on the machine. As before, the cavity was cut according to the simulator film and filled with Cerrobend. For mounting the block to the tray, the index holes of the tray were aligned with the four corresponding indentations in the styrofoam mold that had been made by the attachment spikes of the mold cutting machine. From that point on, we used the same mounting procedure as previously described. A check film (Fig. 5B) shows that the shape of the block has been accurately reproduced, but the position of the block is in error. The error projected to the isocenter is 5.5 mm, in agreement wwiththe 7 mm bend of the tracing wand if the 1.22‐fold film magnification is taken into account.

To further demonstrate the forgiving nature of our block making technique, we laterally offset the support rails for the styrofoam blocks by 8 mm, simulating a poorly aligned mold making machine. In this test we used a straight cutting wand. While the conventional block mounting technique reflected the misalignment, blocks mounted according to our technique accurately matched the simulator film. As a final test, we cut blocks using the misaligned cutting machine and the bent wand. While the conventional mounting method showed large discrepancies between simulator film and check film, blocks mounted with our technique were accurate.

### Clinical performance

To assess the clinical practicality and accuracy of our technique, we used the simulator to take 47 quality assurance check films of beam blocks made for 16 randomly selected patients. These check films were compared to the original simulator films (traditional and computerized tomography‐based virtual simulator) from which the blocks were made. Comparison was done by placing the original simulator film on a light box and the check film on top of it. After arranging the two films for the best match of the block outlines, the axes of the underlying film were traced on the check film using a sharp pencil. The discrepancy between the respective axes was measured with a graduated microscope and used as an indicator for the accuracy of the mounted blocks. Measurements were done separately for the *x* and the *y* errors. The total error was computed as the square root of the sum of the squared individual errors.

**Figure 6:**
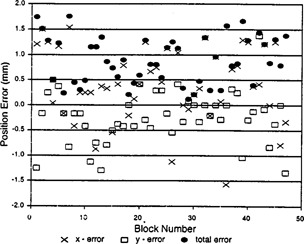
Positional errors of 47 beam blocks.

Figure 6 depicts the position errors for the 47 blocks expressed as the error in a plane through the isocenter. Statistical evaluation of the 47 cases gave values for the *x, y*, and total errors of 0.41±0.72 mm, −0.23±0.52 mm, and 0.89±0.46 mm, respectively. The largest total error was 1.75 mm. The uncertainty of the mean error was ±0.11 for the *x* coordinate, ±0.08 mm for the *y* coordinate, and ±0.07 mm for the total error.

The 0.41 mm average positional error along the *x* direction, being substantially larger than its standard deviation of ±0.11 mm, was evidence for a systematic error. The source of the error was identified as the accessory mount of the simulator that had laterally shifted by 0.3 mm with respect to central ray, causing blocking trays and blocks to be misaligned by the same distance. Taking the 1.54‐fold magnification at the isocenter into account, the small misalignment of the simulator accounted for the observed flaw, implying that the average lateral position of the blocks on the trays was accurate within about 0.1 mm. The small error along the *y* axis, being only −0.15 mm at the position of the shadow tray, was considered insignificant and was not further investigated.

The time spent to make the three indexing marks on each mold, measured with the aid of a stop watch, was 25.2±2.2 seconds; times ranged from 22 to 30 seconds. We could have shortened the time by increasing the temperature of the cutting wire, but this would have compromised the sharpness of the marks.

## CONCLUSIONS AND DISCUSSION

A technique for improving accuracy of custom beam blocks is presented. Laboratory tests demonstrated its capability to compensate for minor imprecisions of the mold making machine. In the clinic, the technique was practical and provided custom blocks with sub mm accuracy. Compared to the previously used traditional method, the extra half minute it took to cut the reference markers was a worthwhile tradeoff for the higher accuracy and ease of operation.

Although it should be possible to achieve comparable accuracy using standard techniques, the demands on the precision of mold making machines, especially older models, would be difficult to meet. Because of the more than 1.5‐fold magnification of beam blocks in the plane through the isocenter, the entire machine, including the tracing wand, would have to be built and aligned with tolerances of no more than a few tenths of a mm. While such accuracy should be achievable for a given configuration, it could be lost each time the machine is readjusted to make blocks from a simulator film that was taken at a different source‐to‐film distance. Replacing a broken cutting wire may also require careful realignment of the wand. In contrast, since we introduced the proposed technique many years ago, the machine is being checked only annually, and realignment is rarely needed.

A block mounting technique that incorporates some features of the proposed method involves making a single marker cut into the mold along the *y* axis, starting at the proximal border and extending to the central ray position. Central ray is marked by two short lateral excursions of the tracing wand along the *x* axis of the simulator film. The merit of this method is that it does not require any modifications of commercial mold making machines. However, the method cannot be applied without modification when blocks involving central ray are made, and the center of the mold is unavailable for the required marker cuts. Depending on the position of the blocks, the deep cut may also affect the rigidity and thereby the precision of the mold. Precision may also be lower, as the marker cut spans only half the width of the mold, and angular alignment errors are “leveraged.” Thus, a small angular misalignment between mold and tray that escapes detection because of the short marker, may lead to an appreciable position error in a block located near the distal border of the mold. In the proposed method, on the other hand, the lateral marker cuts take advantage of the full width of the mold, making rotational misalignment easier to detect.

To achieve accurate block positioning, Johnson and Gerbi^4^ suggest readjusting the blocks after they have been mounted on the tray, using the light field of the simulator. While such a technique compensates for inaccuracies due to the block making machine and human error, it requires access to the simulator and is limited by the precision of the simulator. For example, a poorly adjusted light field in an otherwise precise simulator would erroneously signal an error in a block that is positioned precisely with respect to the radiation field. An imprecise accessory mount of the simulator would also lead to an erroneous block shift. The 0.3 mm error of our simulator would have been propagated instead of being detected.

Adjusting blocks to correct for flaws in the mold making machine raises concerns about the penumbra of the diverging blocks. In the example illustrated in Fig. 1, the block is shifted to correct for a bent stylus and therefore, the point of block convergence is displaced from the focal point of the accelerator. Consequently, the projection of the block surface proximal to the focus is offset with respect to the projection of the distal surface, resulting in a widened penumbra. Using elementary geometry, one can show that the offset distance between the projections is the product of the block shift distance times the difference in magnification between the proximal and the distal surface of the blocks. For our accelerators (65.4 cm tray‐to‐focus distance, 7.5 cm thick blocks), a block shift to correct for a 7 mm error at the isocenter would widen the block penumbra by less than one mm. Penumbra widening is not a unique feature of the proposed method, as the same increase in penumbra width can be expected from any block mounting technique that involves shifting blocks to correct for positioning errors.

A popular method for affixing blocks involves placing the tray on top of the Cerrobend filled mold, inserting screws through existing holes in the tray into the molten metal, and letting the alloy solidify. Since the essential features of our self‐correcting technique are not affected by the specific block attachment method, it should work with this one or any other attachment method. Nevertheless, we have chosen not to use this method since the heat from the molten Cerrobend warps the plastic trays until the alloy has cooled off, casting doubt about accuracy. Research concerning accuracy of this popular mounting method is warranted, as it is very expedient and practical.

## ACKNOWLEDGMENTS

The authors extend their gratitude to Dr. Arnold Feldman for many fruitful discussions. We are also indebted to Adam Woodard for his help with taking simulator check films and to Dr. Sui Shen for a critical review of the manuscript.
